# Technical note: New applications for on-line automated solid phase extraction

**DOI:** 10.1155/S1463924697000199

**Published:** 1997

**Authors:** John D. MacFarlane

**Affiliations:** JM Science, Inc. PO Box 250/355 Lang Blvd Grand Island NY 14072-0250 USA

## Abstract

This technical note explains the disadvantages of manual solid phase extraction (SPE) techniques and the benefits to be gained with automatic systems. The note reports on a number of general and highly specific applications using the Sample Preparation Unit OSP-2A.


Journal of Automatic Chemistry, Vol. 19, No. 5 (September–October 1997) pp. 175–180

### Technical note: New applications for on-line automated solid phase extraction

**John D. MacFarlane**
*JM Science, Inc., PO Box 250/355 Lang Blvd, Grand Island, NY 14072-0250 USA*

*This technical note explains the disadvantages of manual solid phase extraction (SPE) techniques and the benefits to be gained with automatic systems. The note reports on a number of general and highly specific applications using the Sample Preparation Unit OSP-2A.*

### Introduction

Of the various sample preparation methods commonly used, solid phase extraction (SPE) is one of the most important. The main tasks accomplished by solid phase extraction are the separation of interfering matrix components in a sample (clean up); and the enrichment of analytes that occur in biological fluids or in contaminated water in very low concentrations. The following time-consuming steps generally have to be carried out in SPE applications:

(1) Conditioning of the extraction material with various solvents.

(2) Application of sample solution.

(3) Washing steps with solvents or solutions.

(4) Drying of the sorbent.

(5) Elution of the analyte.

(6) Distillation under vacuum or drying with nitrogen.

(7) Dissolution of the residue.

(8) Filtration of the solution.

(9) If necessary, filling and preparing sample vessels.

These manual working steps are expensive in both time and personnel.

### Automatic off-line and on-line SPE

Automation of SPE that otherwise would have to be carried out manually is a considerable help for the user. Such automatic off-line systems, however, have disadvantages as well as advantages. They are not suited to the enrichment of samples from a large sample volume; they are generally open systems (so there is a possibility of contamination); the eluates must be concentrated (so there could be loss through vaporization); or the enriched sample can only be used in diluted form for the subsequent analysis (enrichment loss).

Sample preparation can be considerably improved by on-line automation, which can be integrated into an HPLC system. Small stainless steel or plastic cartridges replace the large cartridges used in manual SPE. The system is completely closed, and the enriched and purified sample can be injected on to the analytical column on-line without loss. Such an instrument is the 'on-line sample preparation unit' incorporating two working positions—the OSP-2A. This instrument can operate simultaneously with two small SPE cartridges.

### Properties and advantages of automatic SPE

If large numbers of samples have to be processed, the analyst will always be interested in automating recurring steps that tend to consume much time and work. Automation of solid phase extraction with the OSP-2A functions as follows:

(*a*) While the sample is being eluted and chromatographed via the second valve of the OSP-2A (analysis side).
(*b*) The cartridge is conditioned and the next sample applied via valve 1 (sample preparation side).
(*c*) In conjunction with an autosampler, the manual steps 1–3 and 5 are carried out fully automatically.
(*d*) Steps 4 and 6–9 are not required due to the on-line procedure used.

This particular type of procedure is a combination of SPE and HPLC. However, it can be included in the list of 'hyphenated techniques' (SPE-HPLC). A block diagram is shown in figure 1.

### Automatic solid phase extraction and its applications

This section describes applications from the pharmaceutical and environmental area. For fully automatic SPE, the sample preparation unit OSP-2A was used.

### *Pesticides*

The pesticide application described here is representative of many other applications with the OSP-2A within the environmental area. Due to legal requirements (TVO, EPA norm), the determination of pesticides in drinking-, ground-, and surface-water is gaining in importance. For example, at least 16 pesticides have to be quantified to a detection limit of 0·1 μg/l. This detection limit can only be achieved if the analyte is enriched from a larger sample volume. Using the off-line technique, a volume of approximately 1000 ml would be required, while with the on-line process 50 ml is sufficient. One example of such an application is the enrichment of 18 pesticides in the 100 ng range, whereby the analytical separation conditions could well be further optimized. Excellent results have been achieved in the investigation of

0142-0453/97 $12·00 © 1997 Taylor & Francis Ltd

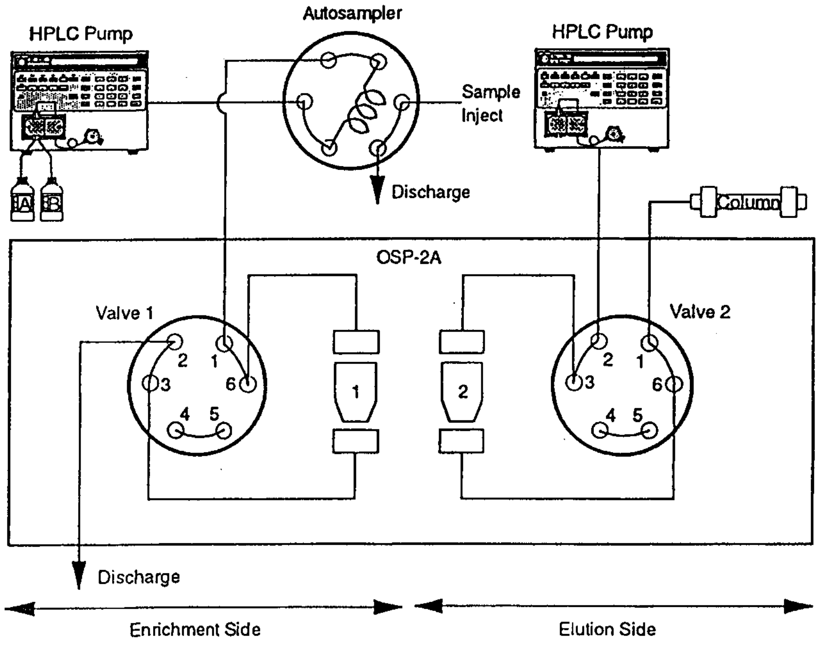

*Figure 1. The plumbing connections in a fully automated HPLC system with on-line sample preparation using OSP-2A.*

drinking- and ground-water containing little or no humic acids. Humic acids have considerable influence on baseline quality and make evaluation more difficult. The screening methods described in the literature reduce, to a large extent, the use of mass spectrometry (MS) for all final investigations as only those positive samples obtained from HPLC screening need to be analysed further.

Excellent recovery rates and reproducibilities can be obtained in pesticide enrichment. The recovery rates were 100%, even with repeated use of the same cartridge. The repeatability was between 0·8 and 5·2% relative standard deviation (RSD).

*Pharmaceutical substances*

Automated SPE with the OSP-2A is frequently used for pharmaceutical analysis, principally for the quality control of active ingredients in drugs, or for the determination of active ingredient concentration in urine, plasma or flesh. A frequent analysis is the decrease of an active ingredient in body fluid as a function of time subsequent to the administration of the medication. As drugs tend to be administered in increasingly smaller doses, the low concentrations of active ingredients must be enriched prior to detection. Simultaneously with such concentration, the analytes can be purified by separation of undesired matrix components.

One interesting application is representative of these examples: the determination of the doping agent stanozolol in plasma. In the case of direct injection of a diluted sample of plasma, stanozolol would not be detectable as numerous UV-active components tend to interfere with the detection of the substance (figure 2). Only through enrichment on a LiChrospher RP-Select-B material, and subsequent separation of matrix components, can quantification be achieved in the 100 ng range. In this optimized application, recovery rates of 99% can be achieved. Excellent reproducibility of ±1·3% RSD demonstrates the high degree of precision of automated solid phase extraction with the OSP-2A. The cartridges can be used repeatedly, subsequent to cleaning without affecting recovery or reproducibility. Automation also saves a great deal of time: samples can be processed automatically overnight or over a weekend.

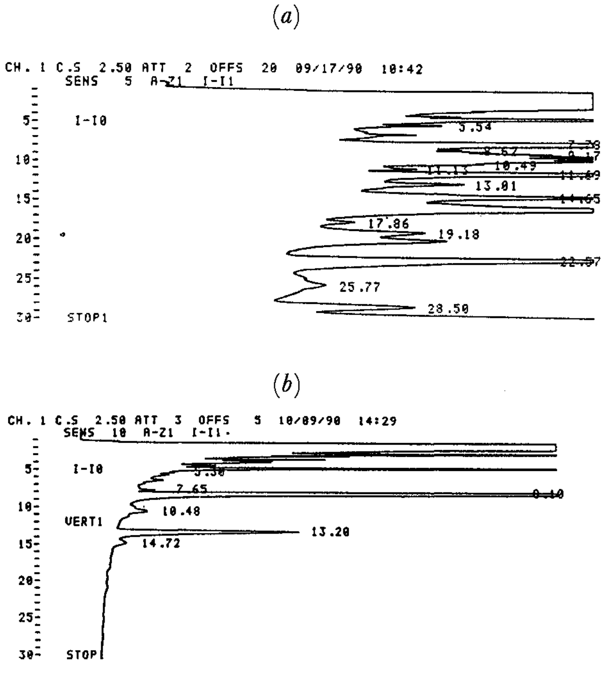

*Figure 2. (a) Chromatogram of a plasma sample without sample preparation step with OSP-2A quantitative determination of the stanozolol peak (RT = 13·02) is not possible. (b) Chromatogram of a plasma sample prepared by an automated solid-phase extraction step using OSP-2A. The quantitative determination of the compound with RT = 13·20 (stanozolol) is now possible. Chromatography: UV: 224 nm; injection volume: 200 µl; 250 ng stanozolol/200 µl; Cartridge: LiChrospher® RP-select B.*

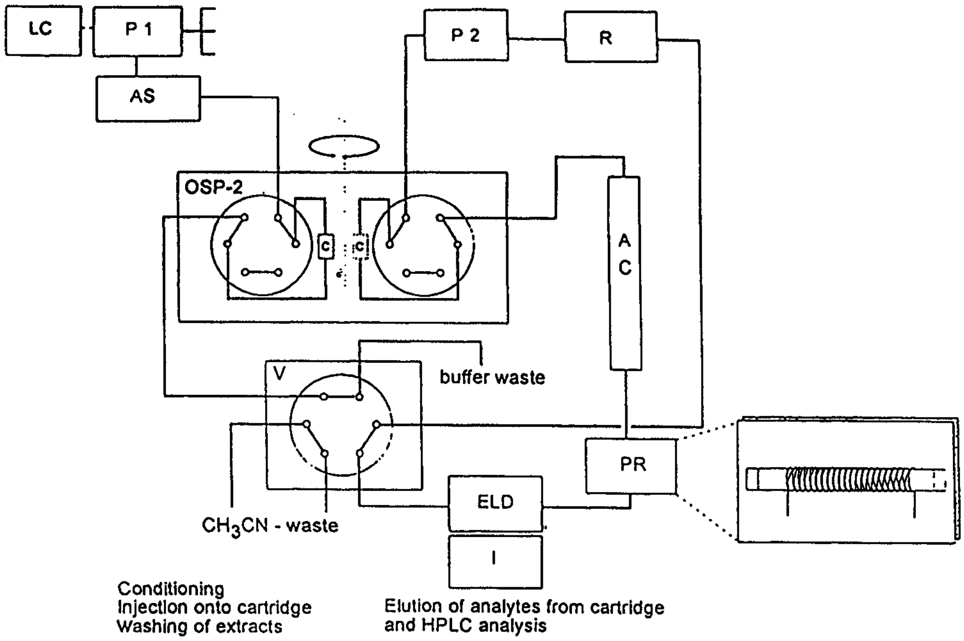

*Figure 3. OSP-2A-HPLC-EC arrangement. AS: Autosampler, AC: column oven and analytical column, C: OSP-2A cartridge, 4×4 mm, ELD: amperometric detector, LC=LC controller, I: integrator, P1=pump 1: LC 655A, P2=pump 2: isocratic pump, PR: photochemical reactor, R: reservoir mobile phase, V: six-port electric valve.*

*Special applications*

The OSP-2A is not only suitable for general daily routine tasks, but also for special applications, for example the highly sensitive detection of penicillin derivatives in bovine muscle with the OSP-2A coupled to an amperometric detector (figure 3).

*On-line coupling of automatic solid phase extraction and electrochemical detection:* Penicillins are used for the treatment of infection in livestock. However, in such cases, residues can contaminate milk and meat. The constant consumption of such contaminated food can damage organs and lead to the production of resistant bacterial strains. For this reason, the European Commission has established limits for the control of food. For benzyl penicillin, the limit for meat is 0·05 mg/kg.

The usual HPLC analytical procedures based on UV/VIS or on fluorescence detection are extremely time-consuming due to the necessary external sample preparation and pre- or post-column derivatization. They are also not sensitive enough.

In the case of pencillin derivatives benzyl penicillin, phenoxymethyl penicillin, oxacillin, cloxacillin and dicloxacillin, an extremely sensitive HPLC procedure is available that combines automatic sample preparation and subsequent photochemical derivatization using electrochemical detection.

Automatic sample preparation in the form of solid phase extraction is carried out with the OSP-2A and included in the HPLC method. This is the first time that the OSP-2A has been successfully combined with the highly sensitive electrochemical detector (ECD). Sensitive quantification is made possible by using a photo-reactor located between the analytical column and the ECD (figure 3).

The OSP-2A cartridges used contain LiChrospher-RP-18e material and can be used a number of times for this application. Checks carried out using penicillin standards resulted in excellent recovery rates of 100%. Detection limits between 1·2 and 4·6 ng were achieved with the standards of the above-mentioned penicillin derivatives. The reproducibility of the method has been optimized in the case of individual derivatives to the extent of 1·4% RSD.

Overall, the combination of the ECD with the OSP-2A is an extremely economical and unsurpassed alternative compared to the time-consuming off-line technique. It enables foodstuffs to be checked quickly and effectively for penicillin residues.

*OSP-2A–MS coupling:* A widespread and important area in environmental research is the determination of polyaromatic hydrocarbons (PAHs) and pesticides along with their metabolites in water and soil. Most PAHs are mutagenic and/or show carcinogenic properties. They are almost insoluble in water and are usually present in very low concentrations.

*Particle beam–mass spectrometry:* In this application, a method is described that enables PAHs to be detected and quantified, even when present in very low concentrations. The coupling of automated SPE using the OSP-2 and HPLC with particle beam-mass spectrometer (PB-MS) (figure 4) makes it easier for the user to make comparisons, and to obtain rapid and reliable identification of the enriched and detected analytes as, especially for PB-MS measurement, a comprehensive data bank is available for reference.

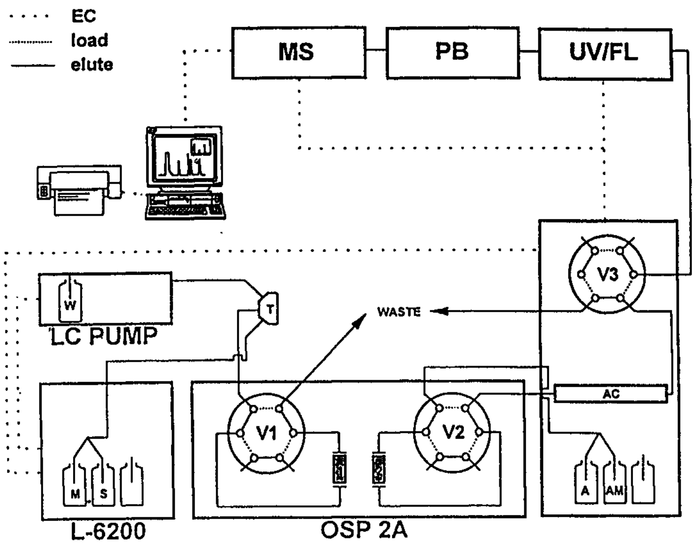

*Figure 4. Schematic representation of the set-up for automated analysis of PAHs. L-6200: gradient pump, OSP-2A: sample preparation unit, LC pump: for delivery of HPLC-grade water for disruption of micelles, T: T-piece, UV/FL: UV detector or FL detector, PB: particle beam interface, MS: mass spectrometer, V1/V2: six-port switching valves of OSP-2A, V3: six-port switching valve of the gradient system, A: acetonitrile, AM: ammonium acetate, M: methanol, S: sample, W: HPLC-grade water, EC: electronical connections, 1/2: positions of the OSP-2A cartridge.*

The well-known problems connected with adsorption in PAH analysis are easily and effectively solved by the addition of a detergent. Using this method, recovery rates of around 100% can be achieved even for the higher homologues of PAHs. The application can be adapted to other HPLC systems and cartridge material. Acids and polar pesticides are widespread, but are, however, difficult to determine as they can only be enriched at low recovery rates. Quantification is thus made more difficult by the presence of humic and fulvic acids. Investigations have shown that the enrichment of pesticides using automated SPE, separation using HPLC and exact identification and quantification using a mass spectrometer can produce excellent results.

*TSP-MS, ESP-MS:* By LC coupling to an electrospray or thermospray mass spectrometer (ESP-MS, TSP-MS), pesticides can be more clearly determined and quantified than previously possible using the diode array detector (DAD). Even those pesticides that possess weak chromophores or whose evaluation is made more difficult by the presence of fulvic and humic acid contaminants in the sample solution can be detected. The detection limit is 0·01–0·03 μg/l and, hence, clearly below the limit of 0·1 μg/l, as prescribed by the European Commission.

Using the method described, 6- and 8-hydroxybentazone can be determined in a concentration range of under 0·1 μg/l. Unequivocal determination in this lower range has not been possible using other methods (figure 5).

The coupling of automated solid phase extraction or sample concentration with HPLC-MS has distinct advantages. The HPLC system can be completely automated, even in the case of the concentration of large sample volumes. Due to the availability of concentrated samples, the MS system no longer operates at its limits of detection. Evaluation and quantification are accurate and precise. Low levels of contamination (humic acids) do not influence the result. Available data banks enable the rapid identification of unknown components.

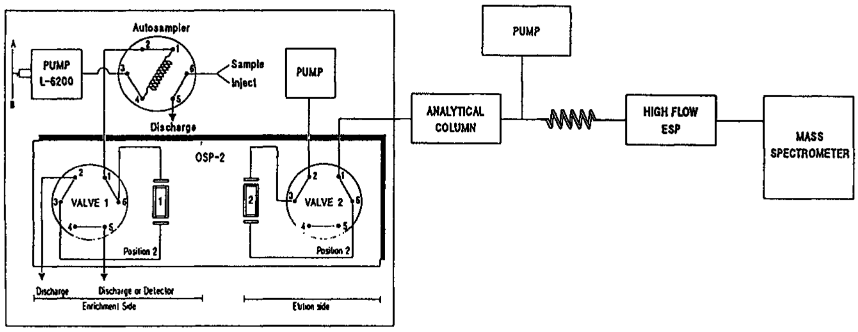

*Figure 5. Schematic diagram of the automated on-line preconcentration by LC postcolumn addition and ESP-MS.*

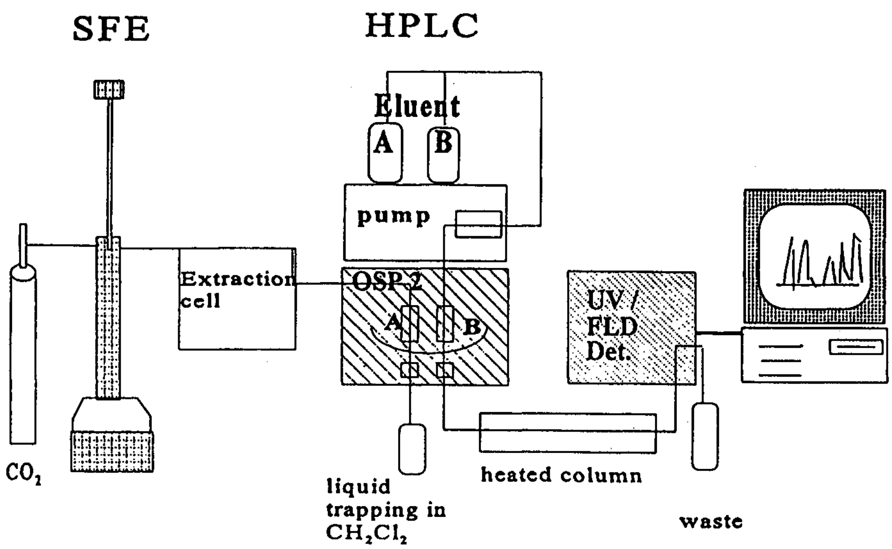

*Figure 6. Schematic representation of the set-up for SFE-SPE-HPLC coupling.*

*SFE–OSP-2A–HPLC:* Another highly interesting application of the OSP-2A is the determination of PAHs in soil. In this application, the automatic sampler is employed as a 'trap' for SFE (supercritical fluid extraction) with subsequent HPLC separation and fluorescence detection (figure 6).

SFE is more suitable than other extraction techniques for the uniform extraction of PAHs from soil or solids. In this technique, the carbon dioxide phase, which is heated to 150 °C and subjected to pressure, is passed through the sample cartridge, and then, once extraction has taken place, through the OSP-2A cartridge (trap) whilst being cooled. The RP-18 material in the OSP-2A cartridge adsorbs the analyte, enriches it and purifies it from hydrophilic matrix components. In a second step, the concentrated analytes in the eluant flow are injected directly onto the HPLC column, separated and quantified using fluorescence detection. This procedure has the advantage of being automated and, hence, eliminates the expensive and time-consuming liquid–liquid extraction step. This detection method is highly sensitive as all extracted analytes can be injected onto the column. Recovery rates are between 90 and 100% This method can also be successfully used for the determination of palladium and rhodium diketonates.

*HPLC–OSP-2–TLC:* In the area of multi-dimensional chromatography of complex samples, HPLC can be coupled with thin layer chromatography (TLC) via the sample preparation unit. By including an SPE step with the OSP-2A, aqueous eluents or buffers can be used as the mobile phase of the HPLC. Through the simultaneous use of two valves in the instrument, the HPLC circuit can be separated from the TLC circuit. The HPLC eluate need not be split. Due to the included SPE step, the concentrated and purified analytes of the pre-selected HPLC fraction can be transferred, free from eluents and salts, to a TLC plate by an automatic applicator using a suitable solvent. This configuration enables the highest possible degree of sensitivity of identification to be achieved (figure 7). In this application, the identification and quantification of 4(5)-methylimidazole (4-MEL) in caramel is described. 4-MEL is a reaction product between sugar and ammonium compounds resulting from the heating process. 4-MEL probably has a toxic effect on the nervous system so that only up to 200 mg/kg is allowed. The HPLC–OSP-2–TLC coupling described is a rapid method for the quantification of 4-MEL. The HPLC chromatogram provides a fingerprint which can be used to identify the type of caramel, while the TLC separation enables the

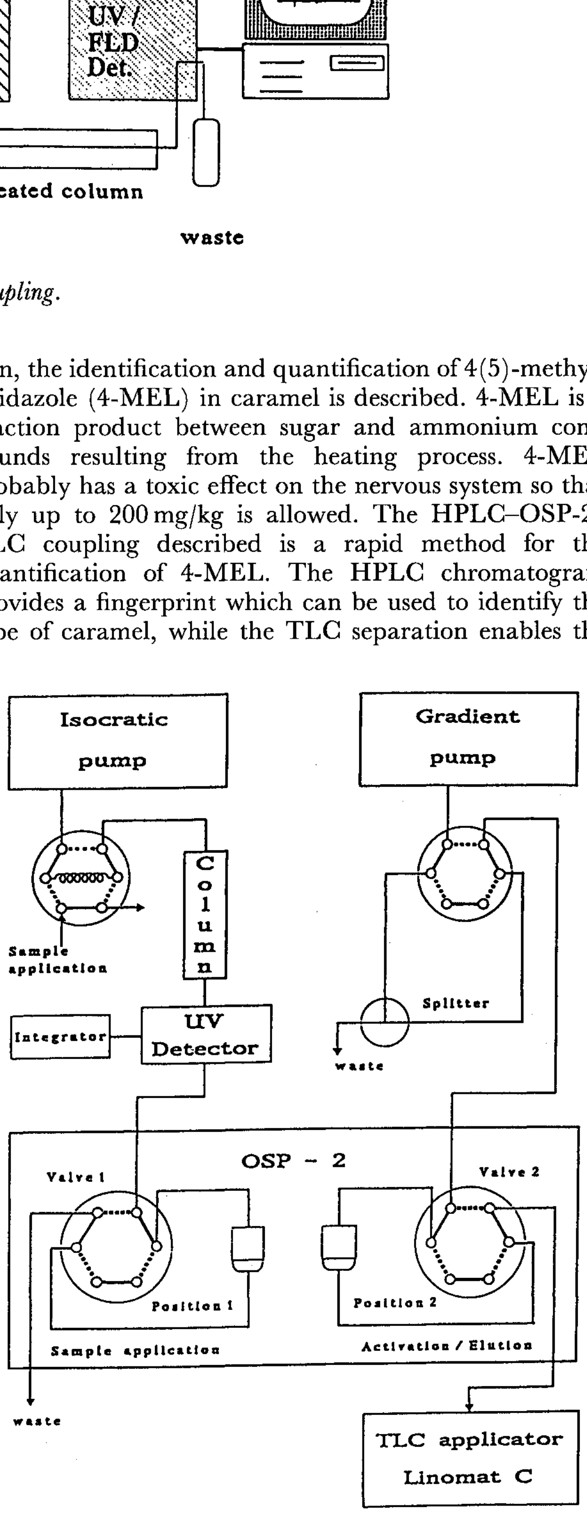

*Figure 7. Instrumental configuration for HPLC-OSP-2-TLC coupling.*

quantity of 4-MEL formed to be determined. The method is particularly suitable for routine investigation and is considerably more efficient than other methods for the determination of 4-MEL. The repeatability of the determination is given as 2·5% RSD. The recovery rates are around 100%, dependent on the type of caramel investigated.

Overall, the method described has numerous advantages over conventional procedures: complete automation of the system, optimal sensitivity of detection, efficient separation of contaminants and interfering salts, optimal selection of application solution for TLC and a reduction of the costs of analysis brought about by time-saving procedures.

### Conclusion

A number of general and highly specific applications using the sample preparation unit OSP-2A have been described. Common to all applications are the efficient automation of purification steps, transfer steps, solid phase extraction and special coupling techniques, all of which help to reduce the costs of analysis and make particularly complex determinations possible. The benefits of the OSP-2A can be summarized as follows:

(1) Elimination of time-consuming manual steps necessary with off-line SPE.

(2) Time saving and higher throughput (on-line SPE-HPLC).

(3) SPE and HPLC operate in parallel.

(4) No contamination (closed system).

(5) No vaporization loss of volatile analytes.

(6) Environmentally friendly through the low volume of solvent required, no evaporation of solvent.

(7) Subjective influences largely excluded.

(8) Highly reproducible results.

(9) No sample loss.

(10) Lower detection limit.

(11) High degree of flexibility with respect to applications.

(12) More rapid analytical results.

(13) Considerable reduction in cost per analysis.

(14) Can be operated around the clock.

In view of the savings being made in the analytical laboratory, automation will continue to be an important topic. Manual sample preparation is a time-consuming and costly procedure. Through automation of the sample preparation steps, the productivity in the chromatographic laboratory can be increased and the quality of analytical results greatly improved

### Further reading

A list of application notes and other papers is available from the author.

